# Characteristics for Low, High and Very High Emergency Department Use for Mental Health Diagnoses from Health Records and Structured Interviews

**DOI:** 10.5811/westjem.18327

**Published:** 2024-02-09

**Authors:** Marie-Josée Fleury, Zhirong Cao, Guy Grenier

**Affiliations:** * McGill University, Department of Psychiatry, Montreal, Canada; † Douglas Mental Health University Research Centre, Montreal, Canada

**Keywords:** emergency department, frequency of emergency department visits, low service users, high service users, very high service users, mental health diagnoses, probability factors, associated variables

## Abstract

**Introduction:**

Patients with mental health diagnoses (MHD) are among the most frequent emergency department (ED) users, suggesting the importance of identifying additional factors associated with their ED use frequency. In this study we assessed various patient sociodemographic and clinical characteristics, and service use associated with low ED users (1–3 visits/year), compared to high (4–7) and very high (8+) ED users with MHD.

**Methods:**

Our study was conducted in four large Quebec (Canada) ED networks. A total of 299 patients with MHD were randomly recruited from these ED in 2021–2022. Structured interviews complemented data from network health records, providing extensive data on participant profiles and their quality of care. We used multivariable multinomial logistic regression to compare low ED use to high and very high ED use.

**Results:**

Over a 12-month period, 39% of patients were low ED users, 37% high, and 24% very high ED users. Compared with low ED users, those at greater probability for high or very high ED use exhibited more violent/disturbed behaviors or social problems, chronic physical illnesses, and barriers to unmet needs. Patients previously hospitalized 1–2 times had lower risk of high or very high ED use than those not previously hospitalized. Compared with low ED users, high and very high ED users showed higher prevalence of personality disorders and suicidal behaviors, respectively. Women had greater probability of high ED use than men. Patients living in rental housing had greater probability of being very high ED users than those living in private housing. Using at least 5+ primary care services and being recurrent ED users two years prior to the last year of ED use had increased probability of very high ED use.

**Conclusion:**

Frequency of ED use was associated with complex issues and higher perceived barriers to unmet needs among patients. Very high ED users had more severe recurrent conditions, such as isolation and suicidal behaviors, despite using more primary care services. Results suggested substantial reduction of barriers to care and improvement on both access and continuity of care for these vulnerable patients, integrating crisis resolution and supported housing services. Limited hospitalizations may sometimes be indicated, protecting against ED use.

Population Health Research Capsule What do we already know about this issue?

*Emergency department (ED) crowding is a major impediment to the efficacy of healthcare systems, caused in part by a minority of patients who use the ED frequently.*

What was the research question?

*We sought to assess patients’ characteristics and service use patterns associated with low, high and very high ED users.*

What was the major finding of the study?

*Violent/disturbed behaviors or social problems increased 5.55 times the probability of very high ED use.*

How does this improve population health?

*A reduction of barriers to care and better access and continuity of outpatient care should be provided for the most vulnerable patients.*

## INTRODUCTION

Emergency department (ED) crowding is a major impediment to the efficacy of healthcare systems,^1^ caused in part by a minority of patients who use the ED frequently.^2^ According to a 2019 systematic review, the estimated prevalence of high ED users was 4-16%, yet these patients accounted for 14–47% of all ED visits, averaging 6.9 ED visits per year.^3^ High ED users, commonly defined as having 4+ ED visits in a 12-month period,^4^ ^,^ ^5^ are more likely than other patients to be hospitalized frequently^6^ and have 2.2 times greater probability of death than other ED users according to a 2015 systematic review.^7^ Mental health diagnoses (MHD), including substance-related disorders (SRD), are very prevalent among high ED users.^1^ ^,^ ^4^ ^,^ ^8^ Another 2013 review reported that between 0.3–18% of patients with MHD were frequent ED users.^8^ A 2019 Canadian study showed that Quebec patients with MHD had used the ED roughly twice as often as patients without MHD, and 17% of these patients were high ED users in 2015-16.^9^ As the ED is not an appropriate setting for treating recurrent patients with MHD, the identification of high ED users and their characteristics is key to improving care among these vulnerable patients and for reducing crowding and healthcare costs in the ED, given that ED use is one of the costliest components of healthcare.^10^

Several studies have assessed patient characteristics associated with high ED use among patients with MHD, most comparing high ED users vs other ED users.^11^ ^–^ ^17^ The sociodemographic characteristics distinguishing high ED users from other ED users included being male,^15^ younger,^14^ single,^16^ having public health insurance,^11^ ^,^ ^12^ and living in more socially or materially deprived^15^ ^,^ ^18^ or metropolitan^15^ areas. Personality disorders,^11^ ^,^ ^13^ ^,^ ^15^ ^,^ ^16^ serious MHD^15^ ^,^ ^17^ or SRD,^5^ ^,^ ^17^ and having chronic physical illnesses^12^ were the main clinical characteristics associated with high ED use. High ED users also differed from other ED users in terms of higher overall use of mental health services.^15^ ^,^ ^19^ ^,^ ^20^ To our knowledge, few studies have compared subgroups of low, high, and very high ED users among patients.^1^ ^,^ ^21^ Those studies have focused on MHD to explain the frequency of ED use, including patients with multiple conditions and with SRD, as the main factor leading to increased use. Very high ED users also reported more recurrent ED use in previous years.^22^ Yet, how the frequency of ED use was categorized differed greatly among these studies: “very high ED use” could be anywhere between 8+^1^ and 18+ visits/year.^21^

A better understanding of patient characteristics associated with low, high, and very high ED users may help tailor interventions and programs to ED profiles and reduce ED use, particularly for high and very high users. We found no previous research comparing low ED users to high and very high users among patients with MHD or SRD. Also, most studies were based solely on single-site hospital health records. Our study is original in that it integrates patient structured interviews with health records from four large mental health networks that include hospitals and community-based services. Very few studies on ED use integrate overall outpatient service use, from primary to specialized care, and assess how these services relate to patient ED use frequency.^22^ Moreover, few studies have tested associations between ED use frequency and quality of outpatient care or motivational behaviors, such as satisfaction with care, unmet needs or perceived stigma that may trigger ED use.

Based on the literature, we hypothesized that very high ED users, followed by high ED users, would be more likely than low ED users to have complex health and social issues and unmet needs, and to use outpatient care more frequently. We assessed various patient sociodemographic and clinical characteristics, and service use patterns associated with low ED users with MHD (1–3 visits/year), compared with high ED users (4–7 visits) and very high ED users (8+ visits) in four large ED networks in Quebec (Canada).

## METHODS

### Description of the Quebec Mental Health System

In Canada, all residents are covered by a universal health insurance managed at the provincial level.^23^ Mental health services, including medication, are mainly public, except services such as psychological services, which are usually paid by the user or covered by some employers. Quebec public healthcare services are mainly managed through 22 large networks, integrating hospitals, long-term and addiction facilities, and community healthcare centers.^24^ In these networks, specialized mental healthcare is provided in psychiatric departments of general hospitals or in psychiatric hospitals, or in specialized addiction treatment centers.^25^ Hospital ED staff include specialized or general emergency physicians, psychiatrists, and psychosocial clinicians—mostly nurses and some social workers and addiction specialists. Primary mental healthcare is offered in medical clinics staffed by general practitioners, in community healthcare centers mainly providing psychosocial services, and by psychologists mostly working in private practice. Community-based organizations, the voluntary sector, integrate crisis and suicide prevention centers, detox centers, and peer support groups.

### Study Settings and Data Collection

The study was conducted in four ED networks serving about two million people—roughly one-fourth of Quebec’s population. Study participants had to be ED users, 18+ years old, able to complete a structured interview, know French or English, and had to grant the research team access to their health records. Study participants were recruited randomly by ED staff based on a health record list of 1,751 ED users who had MHD, including SRD, and had used the ED at least once within the four ED networks in the 12 months preceding recruitment. Of the first 563 eligible patients reached, 450 (80%) agreed to be referred to the research team for consideration as study participants. They were then contacted by the research coordinator and asked to take part in a structured telephone interview, done by trained interviewers closely monitored by the research team.

These interviews were administered between March 1, 2021–May 13, 2022. Average completion time was 45 minutes. Health records for the 12 months prior to interviews were collected to complement interview data, except for previous ED use, which was measured within the two years prior to the last year of ED use. Health records data concerned ED use (Banque de données communes des urgences [BDCU] database), psychiatric outpatient services used, hospitalization (MED-ÉCHO database), and psychosocial services from community healthcare centers (I-CLSC database). Patient diagnoses were included in BDCU and MED-ÉCHO, and framed by the International Classification of Diseases, Canada, 10^th^ Rev (Appendix). All health records included information on patient service use (eg, type, frequency) but exclusively within the ED network. Validated by a steering committee integrating clinicians, structured interview data considered service use outside ED networks and services not included in health records (eg, medical clinics, psychologists). These merged data allowed for a broad dataset on patient service use and other patient characteristics prior to recruitment. Participation in the study was voluntary. Patients who provided consent received a modest financial compensation. The multisite protocol was approved by the ethics review board of the Douglas Mental Health University Institute.

### Study Variables

The dependent variable was ED use frequency for mental health reasons among patients with MHD, measured 12 months prior to interviews. Patients were categorized as low ED users (1–3 visits/year), high ED users (4–7 visits/year) or very high ED users (8+ visits/year). The standard definition of high ED use is 4+ times/year,^11^ ^,^ ^12^ ^,^ ^26^ while very high use was defined as 8+ times/year based on previous^1^ ^,^ ^27^ studies and on a minimal distribution of very high ED visits in the study sample. Independent variables were sociodemographic characteristics, clinical characteristics, and service use patterns, again based on previous research.^21^ ^,^ ^28^

Sociodemographic characteristics included the following: sex; age group; education level; civil status; employment status (eg, worker, unemployed); household income ($Can); type of housing (eg, supervised); number of significant social support network; and stigma. All except “age group” were determined by interview data. Based on the Canadian Community Health Survey (CCHS), social support was measured with the following question: “Do you have one or more people around you on whom you can rely for help with problems? If yes, how many people?” Also based on the CCHS, on a 5-point scale, with responses ranging from “totally disagree” to “totally agree” (greatest stigmatization), stigma was measured with the following affirmation: “Most people in my community treat a person with a MHD or SRD in the same manner as they would treat any other person.”

Clinical characteristics included the following: MHD; SRD; suicidal behaviors (suicide ideation or attempt); violent/disturbed behaviors or social problems; chronic physical illnesses (eg, heart diseases, diabetes); co-occurring MHD-SRD; and high triage priority among ED users. All these variables were based on health records, except SRD, which was based on both health records and the structured interviews. The MHD included serious MHD (schizophrenia spectrum and other psychotic disorders, and bipolar disorders), personality disorders, and common MHD (anxiety, depressive and adjustment disorders; attention deficit/hyperactivity disorder). The SRD integrated alcohol- and drug-related disorders (use, induced, intoxication and withdrawal), measured using health records along with the Alcohol Use Disorders Identification Test^29^ and the Drug Abuse Screening Test-20.^30^ These were included in the structured interviews, as SRD are often underdiagnosed in health records.^31^ We identified chronic physical illnesses and their severity (0 to 2+) based on an adapted version integrating both the Charlson and Elixhauser comorbidity indexes.^32^ The ED triage priority was based on the Canadian Triage Acuity Scale,^33^ consisting of five priority levels or illness severity, with levels 4–5 considered treatable in outpatient care.^33^ In this study, high triage priority ED use (1–3) was considered a proxy for functional disability, based on mean of number of ED visits per patient, with 1–3 triage priority divided by total of ED visits per patient (1–5).

Patient service use included the following: knowledge of mental health or addiction services; having a family doctor or other regular care clinician; frequency of primary care, community-based, and specialized outpatient services used; overall satisfaction with outpatient services used; number of barriers related to unmet needs; frequency of hospitalization, and frequency of previous ED use. Patient service use in the ED networks, mostly mental health specialized care and some primary care services (community healthcare centers), was based on health records, and services outside the ED networks were reported in the structured interviews—mostly primary care, community-based, or specialized addiction services. Service use measured with both types of data integrated only the highest frequency of service use patients reported. As a proxy of continuity of care, patients were asked if they were followed regularly by a family doctor or other clinicians. Based on a previous study,^34^ the benchmark for frequent service use, or minimal intensity of optimal care, was 5+ follow-up appointments/year. Primary care included services received from family doctors, general practitioners in walk-in clinics, psychologists in private practice, and psychosocial clinicians in community healthcare centers.

Community-based organizations integrated crisis and suicide prevention centers, etc. Specialized outpatient care included psychiatric services (eg, treatment from psychiatrist teams, assertive community treatment, and intensive case management programs), and services from addiction treatment centers. Patients were asked to indicate on a 5-point scale their yearly satisfaction with each outpatient service received. We calculated the mean satisfaction score, with higher scores indicating greater satisfaction. Unmet needs were measured through the following CCHS question: “Could you explain the reasons why services outside of the ED did not respond to your needs?” including multiple choice of barriers to care (eg, “I prefer to manage by myself;” “The help is not readily available”). The number of barriers was counted as 0, 1–2, or 3+. Frequency of previous ED use included 4–7 (high ED users) and 8+ ED visits (very high ED users), measured for the two-year period preceding the 12-month interview period.

### Analyses

Missing values (<1%) were imputed by mean for continuous variables and mode for categorical variables.^35^ Descriptive analyses included percentages for categorical variables and mean values for continuous variables. We used bivariate multinomial logistic regression to examine the associations between each independent variable and the dependent variable, frequency of ED use. The intraclass correlation coefficient (ICC) for the study was small (<0.01), indicating low shared variance among patients from the ED networks; multilevel analysis was not required. Based on criterion procedures for forward model selection, independent variables identified as significant in the bivariate analyses (Alpha: 0.20)^36^ were entered sequentially into the multivariable multinomial logistic regression model for frequency of ED use, with low ED use (1–3 visits/year) as the reference group. We used the Akaike Information Criterion (AIC)^37^ to compare the relative goodness of fit among different models before selecting the final multivariate model with the smallest AIC that best fit the data. We also used variance inflation factor (VIF) to measure the amount of multicollinearity in regression analysis and found smaller than 4, indicating that multicollinearity was not a concern.^38^ Relative risk ratios (RRR) and 95% confidence intervals (CI) were calculated in the final model. We performed statistical analyses using Stata 17 (StataCorp LLC, College Station, TX).

## RESULTS

Of the 450 ED users referred, 50 could not be reached and 300 agreed to participate in the study (75% response rate). One patient was withdrawn. Of the 299 patients in the final sample, a majority (55%) were women; 39% were 30–49 years old, 82% single, and 57% unemployed or retired; 47% had a household income of less than CAN$20,000; 57% had post-secondary education, 58% lived in rental housing, and 50% perceived high stigma (Table 1). Over half (57%) had common MHD, 44% serious MHD, 42% personality disorders, 59% SRD, and 45% chronic physical illnesses; 38% had co-occurring MHD-SRD, 54% suicidal behaviors, and 17% violent/disturbed behaviors or social problems. In terms of ED use, 39% were low ED users (1–3 visits/year), 37% high ED users (4–7 visits/year), and 24% very high ED users (8+ visits/year) (Table 2). Nearly half (46%) had poor to fair knowledge of mental health or addiction services; 88% had a family doctor (74%) or other regular care clinician (58%). In the previous year, 58% had used 5+ primary care services, 26% 5+ services from community-based organizations, and 65% 5+ specialized outpatient care. Overall satisfaction with outpatient services averaged 4.02/5; 37% of participants had unmet needs, with 15% identifying 3+ barriers. A majority (56%) were hospitalized, 35% of those 1–2 times, and 39% had been very high ED users over the previous two-year period.

**Table 1.** Sociodemographic and clinical characteristics of patients using the emergency department (N = 299).

Group		Low ED users (1–3 visits/year)		High ED users (4–7 visits/year)		Very high ED users (8+ visits/year)		Total		Bivariate analysis
		117	39.13	109	36.45	73	24.41	299	100	
		n	%	n	%	n	%	n	%	
	Size (N)	mean	SD	mean	SD	mean	SD	mean	SD	*P*-value
Sociodemographic characteristics (measured in the previous 12 months)										
Women^1^		53	45.3	69	63.3	43	58.9	165	55.18	<0.20
Age^2^	18–29 years	30	25.64	36	33.03	26	35.62	92	30.77	<0.20
	30–49 years	48	41.03	41	37.61	28	38.36	117	39.13	
	50+ years	39	33.33	32	29.36	19	26.03	90	30.1	
Education level^1^	High school or less	48	41.03	50	45.87	32	43.84	130	43.48	≥0.2
	Post-secondary education	69	58.97	59	54.13	41	56.16	169	56.52	
Civil status^1^	Single (including separated, divorced, or widowed)	92	78.63	89	81.65	65	89.04	246	82.27	<0.20
	In couple	25	21.37	20	18.35	8	10.96	53	17.73	
Employment status^1^	Worker or student	58	49.57	41	37.61	31	42.47	130	43.48	≥0.20
	Unemployed or retired^3^	59	50.43	68	62.38	42	57.53	169	56.52	
Household income (Can$/year)^1^	0–$19,999	54	46.15	52	47.71	35	47.95	141	47.16	<0.20
	$20,000–$39,999	30	25.64	38	34.86	21	28.76	89	29.77	
	$40,000+	33	28.21	19	17.43	17	23.29	69	23.07	
Type of housing^1^	Private	28	23.93	25	22.94	7	9.59	60	20.07	<0.20
	Rental	63	53.85	63	57.8	47	64.38	173	57.86	
	Supervised^4^	26	22.22	21	19.27	19	26.03	66	22.07	
Number of significant social support network (mean/SD)^1^		3.52	3.19	3.61	5.08	3.63	5.40	3.58	4.51	≥0.20
Stigma^1^	High	56	47.86	56	51.38	37	50.68	149	49.83	≥0.20
	Medium	23	19.66	19	17.43	12	16.44	54	18.06	
	Low	38	32.48	34	31.19	24	32.88	96	32.11	
Clinical characteristics (measured in the previous 12 months)										
Serious mental health diagnoses (MHD)^2^ ^,^ ^5^ ^,^ ^6^		55	47.01	41	37.61	37	50.68	133	44.48	<0.20
Personality disorders^2^ ^,^ ^5^ ^,^ ^6^		31	26.50	52	47.71	44	60.27	127	42.47	<0.20
Common MHD^2^ ^,^ ^5^ ^,^ ^6^		61	52.14	64	58.72	44	60.27	169	56.52	≥0.20
Substance-related disorders^1^ ^,^ ^2^ ^,^ ^5^ ^,^ ^7^ ^,^ ^8^		62	52.99	65	59.63	48	65.75	175	58.53	<0.20
Suicidal behaviors (suicide ideation or attempt)^2^ ^,^ ^5^		44	37.61	63	57.80	54	73.97	161	53.85	<0.20
Violent/disturbed behaviors or social problems^2^		9	7.69	21	19.27	20	27.40	50	16.72	<0.20
Chronic physical illnesses^2^ ^,^ ^5^		38	32.48	48	44.04	50	68.49	136	45.48	<0.20
Severity of chronic physical illnesses^2^ ^,^ ^5^	0	93	79.49	72	66.06	30	41.1	195	65.22	<0.20
	1	15	12.82	18	16.51	27	36.99	60	20.07	
	2+	9	7.69	19	17.43	16	21.92	44	14.72	
Co-occurring MHD-SRD^1^ ^,^ ^2^ ^,^ ^5^ ^,^ ^7^ ^,^ ^8^		35	29.91	43	39.45	35	47.95	113	37.79	<0.20
Percentage of high priority in ED triage^2^	0–33%	19	16.24	20	18.35	9	12.33	48	16.05	≥0.20
	34%–66%	24	20.51	29	26.61	22	30.14	75	25.08	
	67%–100%	74	63.25	60	55.05	42	57.53	176	58.86	

^1^Patient structured interviews. ^2^ *Banque de données communes des urgences* (BDCU, ED database). ^3^The sample was too small to separate unemployed from retired. ^4^Supervised housing included group homes, residential care, supported apartments, etc. ^5^ *Maintenance et exploitation des données pour l’étude de la clientèle hospitalière* (MED-ÉCHO, hospitalization database). ^6^Patients may have more than one MHD. ^7^Alcohol Use Disorders Identification Test (AUDIT). ^8^Drug Abuse Screening Test-20 (DAST-20). Details of diagnostic codes are presented in the Appendix. *ED*, emergency department.

**Table 2.** Service use of patients using the emergency department (N=299).

Service use (measured in the previous 12 months, or other as specified)										
Group		Low ED users (1–3 visits/year)		High ED users (4–7 visits/year)		Very high ED users (8+ visits/year)		Total		Bivariate analysis
		117	39.13	109	36.45	73	24.41	299	100	
		n	%	n	%	n	%	n	%	*P*-value
	Size (N)	mean	SD	mean	SD	mean	SD	mean	SD
Very good to excellent knowledge of mental health or addiction services^1^		59	50.43	63	57.80	39	53.42	161	53.85	≥0.2
Having a family doctor or other regular care clinician^1^ ^–^ ^3^		102	87.18	96	88.07	66	90.41	264	88.29	<0.20
Frequency of primary care service use^1^	0	25	21.37	22	20.18	5	6.85	52	17.39	<0.20
	1–4	29	24.79	32	29.36	14	19.18	75	25.08	
	5+	63	53.85	55	50.46	54	73.97	172	57.53	
Frequency of service use of community-based organizations^1^ ^,^ ^3^	0	68	58.12	51	46.79	29	39.73	148	49.50	<0.20
	1–4	24	20.51	33	30.28	16	21.92	73	24.41	
	5+	25	21.37	25	22.94	28	38.36	78	26.09	
Frequency of specialized outpatient care use^1^ ^,^ ^4^	0	19	16.24	20	18.35	12	16.44	51	17.06	<0.20
	1–4	28	23.93	18	16.51	9	12.33	55	18.39	
	5+	70	59.83	71	65.14	52	71.23	193	64.55	
Overall satisfaction with outpatient services used (mean/SD)^1^		4.18	0.70	3.98	0.77	3.83	0.81	4.02	0.76	<0.20
Number of barriers related to unmet needs^1^ ^,^ ^5^	0	81	69.23	66	60.55	41	56.16	188	62.88	<0.20
	1–2	24	20.51	24	22.02	17	23.29	65	21.74	
	3+	12	10.26	19	17.43	15	20.55	46	15.38	
Frequency of hospitalizations^1^ ^,^ ^6^	0	54	46.15	47	43.12	30	41.1	131	43.81	<0.20
	1–2	50	42.74	37	33.94	18	24.66	105	35.12	
	3+	13	11.11	25	22.94	25	34.25	63	21.07	
Frequency of previous ED use (measured within the 2 years prior to the 12-month period in which interviews were conducted)^1^ ^,^ ^2^	0–3	45	38.46	37	33.94	14	19.18	96	32.11	<0.20
	4–7 (high ED users)	44	37.61	31	28.44	11	15.07	86	28.76	
	8+ (very high ED users)	28	23.93	41	37.61	48	65.75	117	39.13	

^1^See note ^1^below Table 1. ^2^See note ^2^below Table 1. ^3^ *Système d’information permettant la gestion de l’information clinique et administrative dans le domaine de la santé et des services sociaux* (I-CLSC, community healthcare center database). ^4^Psychiatric outpatient services used database. ^5^Based on the CCHS, barriers to care explaining unmet needs were a) I preferred to manage by myself; b) I haven’t gotten around to it yet (eg, too busy); c) I didn’t have enough confidence in the healthcare system or social services; d) I was afraid about what others would think of me; e) I preferred to ask my family or friends for help; f) I am dissatisfied with the quality of services; g) I don’t know how or where to get this kind of help; h) My job interfered with possible treatment (eg, hours of work); i) The help is not readily available; j) I could not afford to pay; my insurance didn’t cover the cost; and k) Services are not offered in my language. ^6^See note ^5^below Table 1. *ED*, emergency department.

We compared variables associated with high or very high ED users with variables among low ED users (Table 3). Women had 1.30 times more probability of being high ED users than men. Patients living in rental housing had 2.09 times more probability of being very high ED users than those living in private housing. Patients exhibiting violent/disturbed behaviors or social problems, or chronic physical illnesses, respectively, showed 2.87 and 1.02 times increase in probability of high ED use, and a 5.55 and 4.95 times greater probability of very high ED use. Patients with personality disorders had 1.06 times greater probability of high ED use, and those with suicidal behaviors, a 1.29 increased probability of very high ED use. Patients with 3+ barriers related to unmet needs had 1.64 and 2.27 times greater probability of being high or very high ED users, respectively. Patients with 5+ primary care services and high recurrent ED use had 2.5 and 1.53 times greater probability of being very high ED users. Patients hospitalized 1–2 times had a reduced probability of 54% for high and 79% for very high ED use, compared with those not hospitalized.

**Table 3.** Estimations of multivariable multinomial logistic regression model on emergency department (ED) visits (reference group: low ED users, 1–3 visits/year).

	High ED users (4–7 visits/year)				Very high ED users (8+ visits/year)			
	RRR*	*P*-value	95% CI*		RRR*	*P*-value	95% CI*	
Sociodemographic characteristics (measured in the previous 12 months)								
Women vs men	2.30	0.007	1.25	4.23	1.48	0.307	0.70	3.16
Type of housing^1^								
Rental vs private	1.43	0.326	0.70	2.94	3.09	0.036	1.08	8.85
Supervised vs private	0.81	0.631	0.34	1.94	2.18	0.200	0.66	7.18
Clinical characteristics (measured in the previous 12 months)								
Personality disorders	2.04	0.039	1.04	4.01	2.26	0.055	0.98	5.18
Suicidal behaviors (suicide ideation or attempt)	1.81	0.063	0.97	3.38	2.29	0.046	1.01	5.16
Violent/disturbed behaviors or social problems	3.87	0.005	1.52	9.85	6.55	0.001	2.26	19.00
Chronic physical illnesses	2.02	0.043	1.02	4.00	5.95	0.000	2.50	14.13
Service use (measured in the previous 12 months, or other as specified)								
Frequency of primary care service use								
1–4 vs. 0	0.97	0.941	0.41	2.31	1.26	0.737	0.33	4.75
5+ vs. 0	0.83	0.641	0.38	1.80	3.51	0.036	1.09	11.35
Number of barriers related to unmet needs^2^								
1–2 vs. 0	1.05	0.892	0.51	2.15	1.13	0.788	0.46	2.76
3+ vs. 0	2.64	0.032	1.09	6.42	3.27	0.028	1.14	9.44
Frequency of hospitalizations								
1–2 vs. 0	0.46	0.037	0.22	0.96	0.21	0.002	0.08	0.56
3+ vs. 0	1.47	0.410	0.59	3.69	1.15	0.797	0.39	3.45
Frequency of previous ED use (measured within the 2 years prior to the 12-month period in which interviews were conducted)								
4–7 (high ED users) vs. 0–3	0.70	0.308	0.35	1.40	0.56	0.788	0.46	2.76
8+ (very high ED users) vs. 0–3	0.93	0.855	0.44	1.97	2.53	0.028	1.14	9.44

*ED*, emergency department; *RRR, relative risk ratio; *CI*, confidence interval. ^1^See note ^4^below Table 1. ^2^See note ^5^below Table 2.

## DISCUSSION

In this study we aimed to identify sociodemographic and clinical characteristics, as well as service use, among patients with MHD, comparing low (1–3 visits/year) to high (4–7 visits) and very high ED use (8+ visits) for mental health reasons. Most patients had high (37%) or very high (24%) ED use, which may be explained by the substantial social and health issues they faced. Their levels of social and material deprivation were high, as was their perceived stigma. Nearly half had serious MHD, personality disorders or chronic physical illnesses, while most experienced SRD and suicidal behaviors. About 40% reported unmet needs or poor to fair knowledge of services, which may explain their high overall ED use. As found in other studies,^13^ ^,^ ^28^ most high ED users were also high users of outpatient care and were frequently hospitalized.

Findings partly confirmed the hypotheses that very high ED users, followed by high ED users, were more likely than low ED users to have complex health and social issues, unmet needs, and to make more frequent use of outpatient care. The result—showing that disturbed/violent behaviors or social problems were the patient characteristics most strongly associated with both very high and high ED use—underlined the special needs of these patients, who for some were likely involuntary ED users. Police are frequently called in to deal with people presenting violent or erratic behaviors and to transport them to ED.^39^

Intervention plans^40^ integrating behavioral treatment^41^ and help in crisis resolution^42^ ^,^ ^43^ may be better deployed for these high and very high ED users. Studies have shown that few overall interventions are being deployed in the ED for high users.^44^ ^,^ ^45^ Previous studies have also shown that patients with chronic physical illnesses made more ED visits.^21^ ^,^ ^26^ Those with co-occurring issues had poorer health overall, higher risk of medication interactions^46^ and more distress,^47^ explaining their frequent ED use. Improving collaborative care^48^ between psychiatrists and primary care services for better treatment of patients with co-occurring issues may also reduce their ED use.

Higher perceived barriers for unmet needs were also strongly associated with more ED use. Barriers may be structural (eg, lack of access to services) or motivational (eg, due to distrust or dissatisfaction with services).^49^ A US study on barriers to care among frequent ED users found that most of them perceived the ED as the only place where their health problems would be treated.^50^ These results highlight the importance of acknowledging barriers to outpatient care and developing more personalized patient care based on recovery-orientated services with patient-centred interventions,^51^ ^,^ ^52^ or alternative “rapid” specialized responses for patients with MHD in crisis.^53^ ^,^ ^54^ Even if very high ED users received primary care more frequently, it doesn’t mean those services were adequate or sufficient to reduce or prevent unmet needs.

Our finding that being hospitalized 1-2 times, but not 3+ times/year, was protective against high or very high ED use compared with not being hospitalized, was an original result. Most hospitalized patients are referred by emergency physicians,^55^ which might suggest that these repeated hospitalized patients have very serious health conditions and that their inpatient care episodes may be unavoidable. Lack of ability to refer (eg, time of day) or possibility to refer (eg, long waiting lists) to outpatient care, lack of mental health support in the ED (eg, brief intervention teams)^56^ ^,^ ^57^ or of comfort in treating patients with more complex MHD profiles in outpatient care might also explain frequent patient hospitalizations. Hospitalization may sometimes be the most appropriate solution for maximizing patient recovery.^58^ For patients with 1–2 hospitalizations/year, close follow-up care,^59^ ^,^ ^60^ which is increasingly recommended following discharge, may have contributed to reducing their ED use. Diversified strategies such as assertive community treatment programs,^61^ home treatment teams,^62^ short-stay crisis units,^63^ and crisis intervention teams^64^ are also increasingly being promoted to help reduce acute care use. Although such interventions remain insufficiently deployed in Quebec, the province’s new Mental Health Action Plan (2022–2026) promises to increase their use.^25^

Compared to low ED users, very high ED users had a higher probability of having suicidal behaviors, while high users showed higher probability of having personality disorders. Previous studies have found associations for both these issues with greater ED use.^13^ ^,^ ^16^ ^,^ ^28^ Considering that healthcare systems tend to respond poorly to crisis situations,^55^ especially those that occur outside regular business hours, the fact that these study participants were very high ED users was not surprising. Greater availability of sustained psychosocial programs in primary care and more specialized crisis and suicidal prevention services^65^ may help prevent ED visits for suicidal behaviors.^66^ Dialectical behavior therapy may also be promoted more extensively to reduce symptoms of personality disorders, borderline personality disorder in particular, as reported in a systematic review.^67^ In general, the ED should not replace outpatient care for vulnerable patients, as their capacity to treat such patients was identified as limited.^68^ ^,^ ^69^

Women had a greater probability of high ED use than men, and patients living in rental housing showed a greater probability of very high ED use than those in private housing. Women reportedly use more health services than men,^70^ which for high ED use contradicted previous studies that found more men were high ED users.^15^ ^,^ ^26^ Because high and very high ED users were differentiated in our study, it may account for this divergent result, with no difference found between women and men in very high ED users. The composition of our study sample could also explain this finding, as a majority of participants recruited randomly by ED staff were women. Concerning patients residing in rental housing, they may experience greater deprivation, including inadequate housing support, compared with those living in private or supervised housing, which may account for their very high ED use. Some type of supportive housing with case management^71^ may help these patients avoid frequent ED use. Difficulty to access outpatient care because of long waiting lists or transportation issues might also explain very high ED use among these patients.

Using 5+ primary care services/year and recurrent high ED use were only associated with very high ED users compared to low ED users, but not high ED users. As for high ED users, studies have identified them as high service users in general,^72^ and as being “recurrent” ED users over several consecutive years.^6^ ^,^ ^28^ Our study added to this literature by specifying that only patients who made at least five primary care appointments in the previous year and eight ED visits in the previous two years had a greater probability of being very high ED users (8+ ED visits/year). The greater use of primary care services among very high ED users may be explained by their higher rates of chronic physical illnesses and the greater severity of these conditions, compared with rates for low and high ED users. Perhaps primary care was not adequate or continuous enough to prevent ED use ^22^ ^,^ ^73^ or to prevent or reduce unmet needs. General practitioners have been shown to lack training or sufficient team capacity to adequately follow up on vulnerable patients with MHD.^74^ ^,^ ^75^ Collaborative care may be more promoted between primary and psychiatric care and team work to reduce ED use and better treat these patients.^76^ ^,^ ^77^

## LIMITATIONS

This study had certain limitations that should be noted. First, there is no consensual definition for low, high, and very high ED use. Different definitions than those chosen here could have led to different findings. Second, the study results were difficult to compare with the literature as most studies have compared high ED use with other ED use. Third, structured interviews may be biased due to the patients’ ability to recall, and the health records that were used reflected service use only within the participating networks. Finally, the diversity of healthcare systems may limit the generalization of the study findings, especially in countries that don’t have public healthcare coverage for deprived populations.

## CONCLUSION

This study was innovative in the way it compared low, high, and very high ED users among patients with MHD in Canada, and by using both patient structured interviews and health records. The findings confirmed that higher ED use was associated with complex patient health issues and higher perceived barriers to unmet needs. Patients with very high overall ED use had the most severe conditions, including greater housing vulnerability and isolation, and more suicidal behaviors. They also used more primary care services, possibly because of their severe chronic physical health conditions.

Recurrent ED use over the years also distinguished very high ED users from low users. By contrast, the risk of high and very high ED use was reduced in patients with 1–2 hospitalizations/year, which underlines the potential benefits and pertinence of hospitalization for some patients. Overall, barriers to care should be reduced and better access and continuity of outpatient care provided for the most vulnerable patients, integrating crisis resolution and supported housing services. This may reduce the number of patients with MHD in the ED, decreasing wait times and improving care in the ED.
