# Supplementary material for: Characteristics for Low, High and Very High Emergency Department Use for Mental Health Diagnoses from Health Records and Structured Interviews
Source: West J Emerg Med. 2024 Feb 9;25(2):144–54. doi: 10.5811/westjem.18327 (PMC11000562; doi:10.5811/westjem.18327)
Supplement: Supplementary file 1 [file wjem-25-144-s001.docx]

**Appendix.** Codes for mental heath disorders including substance-related disorders and chronic physical illnesses according to the International Classification of Diseases, Canada, 10th Rev.

| Diagnoses | *International Classification of Diseases, 10th Rev, Canada* (*ICD-10-CA)* |
| --- | --- |
| Mental health disorders (MHD) ^a^ | |
| *Serious MHD* | |
| Schizophrenia spectrum and other psychotic disorders | F20* (schizophrenic disorders); F22* (persistent delusional disorders); F23 (acute and transient psychotic disorders); F24* (induced delusional disorder); F25* (schizoaffective disorders); F28* (other psychotic disorder not due to a substance or known physiological condition); F29* (unspecified psychosis not due to a substance or known physiological condition); F448 (other dissociative and conversion disorders); F481 (depersonalization - derealization syndrome) |
| Bipolar disorders | F300-F302, F308, F309 (manic episode); F310-F317, F318, 319 (bipolar episode) |
| *Personality disorders* | F600 (paranoid personality disorder); F61 (mixed and other personality disorders); F340 (cyclothymic disorder); F341 (dysthymic disorder); F601 (schizoid personality); F603 (borderline personality disorder); F605 (obsessive-compulsive personality disorder); F604 (histrionic personality disorder); F607 (dependent personality disorder); F602 (antisocial personality disorder); F609 (unspecified personality disorder); F21 (schizotypal personality); F606 (avoidant personality disorder); F608 (other specified personality disorders); F681 (factitious disorder); F688 (other specified disorders of adult personality and behaviour); F69 (unspecified disorder of adult personality and behaviour) |
| *Common MHD* | |
| Depressive disorders | F320- F323 (major depressive disorder, single episode); F328 (other depressive episodes); F329 (depressive episode, unspecified); F330-F334 (major depressive disorder, recurrent); F338 (other recurrent depressive disorders); F339 (recurrent depressive disorder, unspecified); F348 (other persistent mood [affective] disorders); F380, F381 (persistent mood [affective] disorder, unspecified); F388 (other specified mood [affective] disorders); F39 (unspecified mood [affective] disorders); F412* (mixed anxiety and depressive disorder)* |
| Anxiety disorders | F40 (phobic anxiety disorders); F41(other anxiety disorders); F42 (obsessive-compulsive disorder); F45 (somatoform disorders); F48 (other neurotic disorders); F93, F94 (disturbance of emotions specific to childhood and adolescence) |
| Adjustment disorders | F430 (acute stress reaction); F431 (post-traumatic stress disorder); F432 (adjustment disorders); F438 (other reactions to severe stress); F439 (reaction to severe stress, unspecified) |
| Attention deficit/hyperactivity disorder | F900; F901; F908; F909 (attention deficit/hyperactivity disorder) |
| Suicide attempt^a, b^ | X60-Y09, Y870, Y871, Y35-Y36, Y890, Y891 |
| Substance-related disorders^a^ |  |
| Alcohol-related disorders | F101*, F102* (alcohol abuse or dependence); F103, F104* (alcohol withdrawal); F105-F109, K700*-K704*, K709*, G621*, I426, K292*, K852, K860, E244, G312, G721, O354 (alcohol-induced disorders); F100*, T510, T511*, T518, T519 (alcohol intoxication) |
| Cannabis-related disorder | F121, F122 (cannabis abuse or dependence); F123-F129 (cannabis-induced disorders); F120, T407 (cannabis intoxication) |
| Drug-related disorders other than cannabis | F111, F131, F141, F151, F161, F181, F191, F112, F132, F142, F152, F162, F182, F192 (drug abuse or dependence); F113-F114, F133-F134, F143-F144, F153-F154, F163-F164, F183-F184, F193-F194 (drug withdrawal) F115-F119, F135-F139, F145-F149, F155-F159, F165-F169, F185-F189, F195-F199 (drug-induced disorders); F110, F130, F140, F150, F160, F180, F190, T400-T406, T408, T409, T423, T424, T426, T427, T435, T436, T438, T439, T509, T528, T529 (drug intoxication) |
| Chronic physical illnesses^a,^ ^c^ | |
| Renal failure | I120, I131, N18, N19, N250, Z49, Z940, Z992 |
| Cerebrovascular illnesses | G45, G46, I60-I69 |
| Neurological illnesses | G10–G12, G13, G20, G21–G22, G254, G255, G312, G318, G319, G32, G35, G36, G37, G40, G41, G931, G934, R470, R56 |
| Endocrine illnesses (hypothyroidism; fluid electrolyte disorders and obesity) | E00, E01, E02, E03, E890; E222, E86, E87; E66 |
| Any tumor with or without metastasis (solid tumor without metastasis; lymphoma) | C00–C26, C30–C34, C37–C41, C43, C45-C58, C60–C76, C77-C79, C80; C81-C85, C88, C900, C902, C96 |
| Chronic pulmonary illnesses | I278, I279, J40-J47, J60-J64, J65, J66, J67, J684, J701, J703 |
| Diabetes complicated and uncomplicated | E102-E108, E112-E118, E132-E138, E142-E148; E100, E101, E109, E110, E111, E119, E130, E131, E139, E140, E141, E149 |
| Cardiovascular illnesses (congestive heart failure; cardiac arrhythmias; valvular illnesses; peripheral vascular illnesses; myocardial infarction; hypertension and pulmonary circulation illnesses) | I099, I110, I130, I132, I255, I420, I425–I429, I43, I50, P290; I441–I443, I456, I459, I47–I49, R000, R001, R008, T821, Z450, Z950; A520, I70-I72, I730, I731, I738, I739, I771, I790, K551, K558, K559, Z958, Z959; I05–I08, I091, I098, I34–I39, Q230–Q233, Q238, Q239, Z952, Z953, Z954I210-I214, I219, I220, I221, I228, I229, I252; I101, I100, I11, I1500, I1501, I1510, I1511, I1521, I1581, I1590, I1591, I674; I26, I27, I280, I288, I289 |
| Other chronic physical illness categories (blood loss anemia; ulcer illnesses; liver illnesses; AIDS/HIV; rheumatoid arthritis/collagen vascular illnesses, coagulopathy; weight loss, paralysis; deficiency anemia) | D500; K257, K259, K267, K269, K277, K279, K287, K289; B20-B24; D65–D68, D691, D693-D696; B18, I85, I864, I982, K700- K703, K709 K711, K713–K715, K716, K717, K721, K729, K73, K74, K754, K760, K761, K763, K764, K765, K766, K768, K769, Z944; L900, L940, L941, L943, M05, M06, M08, M120, M123, M30, M31, M32–M35, M45, M460, M461, M468, M469; G041, G114, G80, G81, G82, G83; E40–E46, R634, R64, D51–D53, D63, D649; D501, D508; D509 |

^a^The ICD-10-CA was used in MED-ÉCHO (*Maintenance et exploitation des données pour l’étude de la clientèle hospitalière*, a hospitalization database) and in BDCU (*Banque de données communes des urgences*, an emergency department (ED) database). Diagnoses related to the two databases were considered, and all data were integrated for the studied period, for each patient. MED-ÉCHO includes several diagnoses: primary diagnosis and numerous secondary diagnoses. For the databases used in this study, MHD were considered as primary diagnoses only, but substance-related disorders (SRD) as both primary and secondary diagnoses, considering that SRD are often underdiagnosed. ^b^Diagnostic codes for suicide attempt were registered in the MED-ÉCHO database. ED use for reasons of suicide ideation or attempt were reported by triage nurses in ED and registered in the BDCU database. As they are not diagnostic codes, they were not reported in this table. ^c^The list of chronic physical illnesses is based on an adapted and validated version of the Elixhauser Comorbidity Index, integrating the Charlson Comorbidity Index, which consists of 32 major categories of physical illnesses (see reference in the Methods section). For this list of chronic physical illnesses, three categories of MHD and two categories of SRD (identified with an asterisk [*]) were also included under MHD-SRD, thus appearing twice.
